# Defect-Intent Ambiguity Addressing for Training-Free Deterministic PCB Defect Localization via Template Selection and Dissimilarity Mapping

**DOI:** 10.3390/s26051541

**Published:** 2026-02-28

**Authors:** Saiyan Saiyod, Woottichai Nonsakhoo, Zhengping Li, Piyanat Sirisawat

**Affiliations:** Hardware-Human Interface and Communications Laboratory (H2I-Comm Lab), Department of Computer Science, College of Computing, Khon Kaen University, Khon Kaen 40002, Thailand; nonsakhoo@cassia.kku.ac.th (W.N.); zhengping.l@cassia.kku.ac.th (Z.L.); piyanat_siri@cassia.kku.ac.th (P.S.)

**Keywords:** printed circuit board (PCB), automated optical inspection (AOI), defect localization, defect detection, image processing, template selection, structural similarity, difference map, correlation, quantile thresholding

## Abstract

Automated optical inspection (AOI) for printed circuit boards (PCBs) requires localizing small, sparse defects under illumination drift and minor placement misalignment, while supporting fast, auditable pass/fail decisions. This paper presents a training-free, reference-based digital image processing framework with no learning/training stage that compares each defective query image with a small library of defect-free reference templates (for the same PCB layout/revision) using a small set of interpretable control parameters. A reference is selected by coarse-to-fine matching (fast pre-screening followed by SSIM refinement on a central region), and an optional global alignment is applied only when it increases SSIM to limit defect-driven over-correction. Defects are highlighted by a defect-likelihood field that fuses an SSIM-derived structural dissimilarity map with a normalized absolute-difference map, followed by connected-component extraction to produce confidence-ranked bounding boxes. The method achieves Precision = 0.9663, Recall = 0.9987, and F1 = 0.9822 at the best-F1 operating point (0.149 false positives per image). Under the adopted box-matching protocol, average precision reaches 0.984. Precision–recall and FROC curves are reported to support threshold selection under different false-alarm budgets.

## 1. Introduction

Automated optical inspection (AOI) is a core quality fcontrol stage in printed circuit board (PCB) manufacturing, where decisions must be made reliably at production speed [1,2]. In practice, true defects are sparse and localized (e.g., missing copper, spurious copper, scratches, and pinholes), yet benign variability in illumination and minor board-placement changes is common [3]; therefore, a useful inspection method must suppress nuisance variability while remaining sensitive to subtle, localized structural deviations. AOI is typically positioned inside a broader in-line quality strategy that aims to reduce scrap and rework while keeping false alarms manageable in production [4].

In many industrial quality control (QC) workflows, the primary decision is pass/fail (accept/reject): if any verified defect is present, the board is rejected. Therefore, reliable defect localization and evidence are central, while fine-grained defect-type classification (e.g., Missing_hole, Mouse_bite, Short) is often optional and can be treated as a downstream step for reporting, process monitoring, or root-cause analysis [5]. At the same time, surveys of PCB defects and inspection practices highlight that defect taxonomies and visual manifestations can vary across processes and products, which complicates universal classification claims and emphasizes the value of interpretable evidence [6].

A key practical disparity is that the notion of a “defect” can be ambiguous without context from the intended design [7]. In PCB engineering, many localized copper features are introduced deliberately to satisfy electromagnetic interference and compatibility (EMI/EMC) and signal/power integrity (SI/PI) constraints rather than to “look clean” in an image [7,8,9,10]. For example, an apparent copper bridge may be an unintended short in one location but an intentional connection, net-tie, or controlled strap in another. Likewise, patterns that resemble spurious copper in isolation can be purposeful copper pours and fills (e.g., ground pours for shielding and controlled return-current paths), via stitching and via fences to reduce radiation and crosstalk, guard traces, copper thieving/balancing for manufacturability, or local tuning structures used to manage impedance, ringing, and high-frequency noise.

Spur-like features and short stubs can also be intentional (e.g., test access, reinforcement/teardrops, thermal relief, or current-spreading structures), even though similar geometries may be considered defects in other contexts. Because these intent-driven features may be small and localized, appearance-only inspection can confuse design intent with anomaly [6,11].

This defect-versus-intent ambiguity motivates reference-based inspection: comparing a query board against a defect-free exemplar of the same layout provides the necessary design-context baseline [12]. The template encodes the intended routing, copper distribution, and layout-specific structures used for EMI/EMC and SI/PI, allowing the inspection decision to focus on deviations from the intended reference (true defects) rather than on visually unusual but intentional patterns [7,8].

In production, the primary constraint is often not peak accuracy in a curated benchmark but predictable behavior under everyday drift and change [4,13]. Practical AOI systems are judged by time-to-deploy on a new board, validation burden, compute footprint, and the ability to explain failures when they occur [13]. End-to-end learning-based models are widely used; however, in some deployment settings their internal decision logic can be harder to audit and their lifecycle cost can include labeling, retraining, monitoring, and re-qualification [5,14]. When an inspection decision is disputed, it is valuable to attribute the response to concrete causes such as illumination shift, residual misalignment, template mismatch, or a true localized defect [15,16].

Accordingly, a recurring deployment theme is to reduce manual labeling burden and model maintenance effort while still improving robustness, which motivates both training-free pipelines and low-label learning regimes [17,18].

These constraints motivate a complementary design point: analysis-first, physics- and mathematics-grounded image processing that exposes its decision signal. Classical industrial inspection continues to rely on deterministic components such as thresholding, morphology, and template matching because they are auditable and controllable [19,20]. In this view, a defect-free template functions as a measurement reference standard: inspection becomes change detection against the intended layout rather than category recognition.

The main obstacle is that simple subtraction is sensitive: small geometric mismatch and brightness drift can dominate the difference image and produce widespread false positives [15]. Robust reference-based inspection therefore benefits from structural similarity measures and careful handling of alignment [21]. The structural similarity index (SSIM) [22] emphasizes structure rather than raw intensity and can be evaluated locally to form an SSIM map that highlights localized changes. Alignment can further reduce nuisance differences, but in defect localization it must be used carefully because defects can bias the estimated transform; a pragmatic safeguard is to accept alignment only when it yields a measurable similarity improvement [23].

Related work on PCB AOI spans broad surveys and focused PCB-specific reviews that summarize classical image processing pipelines and, more recently, deep learning-based approaches [1,2,6,11,14,24]. Classical AVI/AOI surveys establish the foundational problem setting and recurring algorithmic motifs (preprocessing, registration/matching, and post-processing for decision rules) [25]. More recent reviews focus on electronics manufacturing and PCB inspection specifically, including the rise of deep learning and the associated practical considerations (data requirements, interpretability, and lifecycle/maintenance cost) [11,14,24]. Across these perspectives, a consistent theme is that production deployment often rewards predictable behavior and actionable diagnostics, which keeps reference-based inspection relevant even when learning-based models are available.

Beyond RGB surface imagery, inspection and monitoring can also involve specialized imaging and task-specific pipelines, for example in solder-joint and electronic assembly defect analysis, where the sensing modality and measurement objective differ from bare-board surface AOI [26,27].

A long-standing and still common formulation is reference/template-based change detection, where the query is compared to a defect-free exemplar and differences are extracted using similarity measures and post-processing [12,20,21]. In this family, normalized cross-correlation and related similarity measures are widely used to compare a query against a template, both for alignment/matching and for highlighting likely change regions [12,28]. Because AOI images can differ by small shifts, rotations, and illumination drift, many works explicitly study robustness of similarity/dissimilarity measures and the sensitivity of subtraction-based signals to misregistration [15,29]. Feature-based matching and alternative similarity measures (beyond raw subtraction) are also widely explored as ways to increase tolerance to nuisance variation while preserving localized change cues [16,30,31]. Rotation or uncertainty-aware designs further emphasize that handling geometric variability is a practical requirement rather than a corner case [32]. This practical issue is directly aligned with our use of structural similarity and alignment safeguards: the goal is to suppress broad, nuisance-driven responses while preserving localized defect evidence [22,23].

Within classical (training-free) inspection, several works explicitly combine subtraction/matching with deterministic operators for categorization and reporting [19,21,33]. For example, an algorithmic scheme for concurrent detection and classification applies fuzzy c-means segmentation after image subtraction and then uses arithmetic/logic operations, the circle Hough transform, morphological reconstruction, and connected-component labeling to assign defect categories; it reports 100% detection and 99.05% classification accuracy in its experimental setting [33]. In a similar spirit, other subtraction-based pipelines target both detection and defect-type decisions by combining difference images with hand-designed operators (e.g., morphology, logical operations, and component analysis), illustrating the long-standing appeal of deterministic, auditable processing in PCB AOI [19].

In parallel, deep learning has been widely adopted for PCB defect detection and classification, commonly borrowing generic object-detection backbones and adapting them for small, low-contrast defects and industrial constraints [24,34,35,36,37]. One line of work applies one-stage object detectors (often YOLO-family variants) and proposes architectural changes to improve localization of tiny PCB defects while keeping inference efficient [38,39,40,41,42,43,44]. Additional one-stage variants integrate backbone and attention refinements for feature reuse and improved localization under clutter [45,46,47,48]. Another line adapts two-stage and feature-pyramid designs (e.g., Faster R-CNN/FPN-style components) to improve multi-scale defect detection where targets are small and the background is cluttered [49,50,51]. Beyond detector backbones, several studies emphasize lightweight deployment, attention/feature-fusion refinements, and context modeling to trade off accuracy and throughput in practical settings [52,53,54]. Deep learning is also used for related PCB inspection tasks including defect classification, reconstruction/autoencoder-based inspection signals, and component-level understanding such as PCB segmentation for recognition [55,56,57]. Transformer- and DETR-inspired detectors are another recent direction, motivated by global context modeling and end-to-end detection design [58,59]. Beyond standard detector framing, learning-based approaches also include alternative objectives and cues (e.g., energy-based or edge-guided signals), domain adaptation strategies to handle distribution shift [60], and PCB-specific designs for bare-board defects [61,62,63].

Because labeled defect samples can be scarce and production distributions can drift, recent work also explores semi-supervised, unsupervised, and few-shot settings for PCB inspection [17,18,64]. These directions aim to reduce annotation burden and improve adaptability by leveraging unlabeled data, uncertainty modeling, or meta-learning-style transfer from limited support examples [17,18,64].

An important practical gap in PCB inspection is defect-versus-intent ambiguity. Appearance-only cues can confuse intentional layout features with true defects unless a layout-specific reference is available. Motivated by this, this work presents a training-free, reference-based image processing framework that retains the operational advantages of template inspection while improving robustness to everyday nuisance variation. While end-to-end learning-based defect detectors are widely used in industrial inspection across product domains, the proposed pipeline provides an auditable and controllable alternative that does not require a training lifecycle [65,66]. The method is computationally efficient and operationally transparent: it produces interpretable intermediate fields (similarity and difference maps) and relies on controllable operators whose behavior can be inspected and tuned without retraining.

The main contribution of this work lies in a training-free framework design that combines classical operators with explicit robustness safeguards targeting defect-versus-intent ambiguity under everyday nuisance variability. In particular, we emphasize ROI-based template selection, SSIM-gated alignment acceptance, and a fused defect-likelihood mapping that yields confidence-ranked detections for deployment-oriented PR/FROC threshold selection. The key contributions are as follows:

- Coarse-to-fine reference selection with central-region similarity: a fast pre-screening stage plus SSIM refinement computed on a central region to suppress border/outlier effects, with caching and downscaling for throughput.
- SSIM-gated alignment acceptance: an explicit validation rule that applies a global warp only when SSIM improves on the same central region, mitigating defect-driven over-correction.
- Fused, auditable defect-likelihood and operational evaluation: an explicit defect-likelihood field that fuses structural dissimilarity and normalized absolute difference with central-region-based rescaling for stable thresholding, producing confidence-ranked candidates that are characterized via PR/FROC and IoU-sensitivity rather than a single tuned threshold.

The remainder of this paper is organized as follows: Section 2 details the proposed method, Section 3 presents the experimental results and analysis (including the evaluation protocol), Section 4 discusses limitations and practical considerations, and Section 5 concludes this paper.

## 2. Proposed Methods

**Figure 1:**
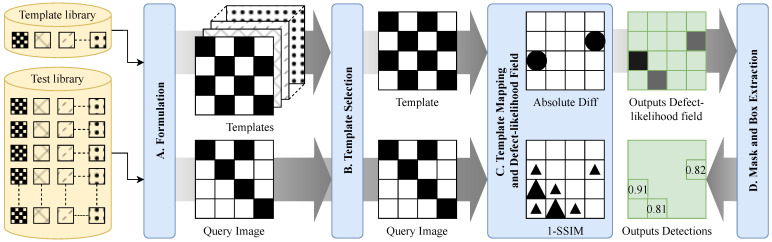
Overview of the proposed image processing framework for PCB defect localization.

This section presents a training-free PCB defect-localization pipeline. For each query image, it selects a defect-free reference template and outputs (i) an interpretable defect-likelihood field and (ii) confidence-ranked bounding boxes. At a high level, the pipeline performs coarse-to-fine template selection, constructs the defect-likelihood field by fusing SSIM-based structural dissimilarity with normalized absolute difference, applies global alignment only when it improves SSIM, and extracts sparse candidates via quantile thresholding, morphology, and connected-components analysis (Figure 1).

### 2.1. Formulation

This work considers reference-based PCB inspection in which, for each defective query image, one or more defect-free exemplars of the same PCB layout are available. Let *I* denote a defective query image and $T={\left\{T_{k}\right\}}_{k=1}^{K}$ denote a library of defect-free templates. Here, templates refer to defect-free reference images for the same PCB layout/revision (not defect-type templates that enumerate defect categories). Consequently, the localization stage is class-agnostic across defect categories: any defect that manifests as a localized deviation from a valid defect-free reference can yield a response in the defect-likelihood field.

The goal is to output the following: (i) a spatial defect-likelihood field M(u,v) (higher values indicate more likely defect) and (ii) a set of *N* axis-aligned bounding boxes with confidence scores $P={\left\{\left(b_{i},s_{i}\right)\right\}}_{i=1}^{N}$, where each $b_{i}=\left[x\;y\;w\;h\right]$ denotes the top-left corner (x,y) and the box width/height (w,h), and $s_{i}$ is a confidence score.

To stabilize similarity computation and threshold estimation, statistics are evaluated on a central image region Ω parameterized by a margin with *m* pixels on all sides. The margin *m* is computed from an area-avoidance ratio p∈[0,1), which specifies the fraction of the total image area to ignore.

### 2.2. Template Selection

All images are converted to grayscale and normalized to [0,1] by an affine intensity rescaling. Scalar SSIM is denoted by SSIM(·,·) [22]. Pixel coordinates are (u,v) (column, row) and the image domain is X={1,…,W}×{1,…,H}.

Similarity and thresholds are evaluated on an inner region Ω parameterized by a symmetric margin *m* determined from an area-avoidance ratio p∈[0,0.95], where 0.95 is an experimental upper limit to ensure a nontrivial inner region. With a margin *m* on all four sides, the central area is (W−2m)(H−2m) and the remaining area is

(1) $$WH-\left(W-2m\right)\left(H-2m\right)=2m\left(W+H\right)-4m^{2}.$$

Setting this equal to pWH yields the (smaller) quadratic root

(2) $$m=\frac{\left(W+H\right)-\sqrt{{\left(W+H\right)}^{2}-4pWH}}{4},$$

and ⌊m⌋ is used as an integer padding. The inner region is

(3) Ω={(u,v)∈X:1+m≤u≤W−m,1+m≤v≤H−m}.

In implementation, this continuous expression is converted to an integer ROI with explicit rounding and safety clamping; see Appendix A.1.

Given a query image *I* and template library ${\left\{T_{k}\right\}}_{k=1}^{K}$, the template is selected by maximizing SSIM on the inner region at a designated selection resolution. Let ${↓}_{D}\left(\cdot \right)$ denote isotropic resizing to satisfy max(H,W)≤D and let N(·) denote grayscale+normalization. At selection scale $D_{\mathrm{sel}}$,

(4) $$I_{\mathrm{sel}}=N\left({↓}_{D_{\mathrm{sel}}}\left(I\right)\right),\;T_{k,\mathrm{sel}}=N\left({↓}_{D_{\mathrm{sel}}}\left(T_{k}\right)\right).$$

To reduce computation, similarity is evaluated only within Ω. Denoting cropping by $C_{\Omega }\left(\cdot \right)$, the selection objective is

(5) $$k^{*}=\arg\max_{k\in \{1,\ldots ,K\}}\;\mathrm{SSIM}\left(C_{\Omega }\left(I_{\mathrm{sel}}\right),\;C_{\Omega }\left(T_{k,\mathrm{sel}}\right)\right),$$

and the chosen template is $T^{*}=T_{k^{*}}$.

To reduce selection time, a two-stage scheme is used: (i) a cheap preselect score at a smaller max dimension $D_{\mathrm{pre}}$ to shortlist $K_{s}$ candidates and (ii) SSIM refinement computed only on that shortlist. Two suitable preselect scores are as follows:

(6) $$\begin{array}{cc} \mathrm{corr}(a,b) & =\frac{\langle a-\bar{a},\;b-\bar{b}\rangle }{∥a-\bar{a}{∥}_{2}\;{∥b-\bar{b}∥}_{2}+\epsilon }, \end{array}$$

(7) $$\begin{array}{cc} \mathrm{mad}(a,b) & =-\frac{1}{\left|\Omega \right|}\sum_{(u,v)\in \Omega }\left|a\left(u,v\right)-b\left(u,v\right)\right|, \end{array}$$

where a,b are vectorized values in Ω after mean subtraction (corr) or are directly compared (mad). Correlation-style matching is a standard building block for template-based inspection and fast preselection [12,28,67]. Further details and implementation considerations (including complexity, shortlist logic, and caching of preprocessed templates to amortize decoding/resizing across queries) are provided in Appendix A.2.

### 2.3. Template Mapping and Defect-Likelihood Field

After selecting $T^{*}$, a defect-likelihood field M(u,v) is constructed on a processing grid. To control runtime, expensive stages (alignment, SSIM-map, and morphology) may be downsampled by selecting a target long-side processed length $D_{\mathrm{proc}}$ and defining an isotropic scale

(8) $$s=\min\left(1,\frac{D_{\mathrm{proc}}}{\max(H_{I},W_{I},H_{T},W_{T})}\right).$$

This yields

(9) $$I_{s}=N\left({↓}_{s}\left(I_{\mathrm{sel}}\right)\right),\;T_{s}=N\left({↓}_{s}\left(T^{*}\right)\right).$$

If needed, the query is resized to match the template grid. The implementation also supports resolution presets and scales morphology/area parameters consistently with the processing scale; see Appendix A.3.

Although the proposed framework can operate without explicit alignment, in practical AOI settings a lightweight global alignment is highly recommended whenever small placement shifts or mild rotations are expected. Because the defect-likelihood field is computed pixel-wise, even minor global misalignment can produce widespread responses that resemble false defects.

Let $\tau_{\theta }$ denote a parametric global warp (translation or rigid) with parameters θ, and let the warped query be $I_{s}\circ \tau_{\theta }$. The alignment is estimated by maximizing an intensity-based similarity inside the inner region Ω,

(10) $$\hat{\theta }=\arg\max_{\theta }\;\rho \left(M_{\Omega }\left(I_{s}\circ \tau_{\theta }\right),\;M_{\Omega }\left(T_{s}\right)\right),$$

where ρ is a correlation-like similarity and $M_{\Omega }$ denotes inner-region masking, i.e., pixels outside Ω are set to zero. More general geometric matching formulations exist (e.g., via epipolar-geometry constraints), but a lightweight global warp is sufficient under the fixture-controlled imaging assumed in AOI lines [68].

Because defect content can bias the estimated warp, the transform is not applied unconditionally. Instead, the candidate warp is validated using SSIM gain on the inner region,

(11) $$\begin{array}{cc} \Delta & =\mathrm{SSIM}(M_{\Omega }\left(I_{s}\circ \tau_{\hat{\theta }}\right),M_{\Omega }\left(T_{s}\right)) \end{array}$$

(12) $$\begin{array}{cc} \; & -\mathrm{SSIM}(M_{\Omega }\left(I_{s}\right),M_{\Omega }\left(T_{s}\right)). \end{array}$$

The alignment is accepted if $\Delta \geq \Delta_{\min}$ (a user-controlled safeguard); otherwise it is rejected. Accordingly, the aligned (or unaligned) query is defined as

(13) $$\tilde{I}=\left\{\begin{array}{cc} I_{s}\circ \tau_{\hat{\theta }}, & \Delta \geq \Delta_{\min},\\ I_{s}, & \mathrm{otherwise}. \end{array}\right.$$

This acceptance rule makes alignment a practical robustness aid while limiting over-correction in the presence of true defects.

To prevent outliers outside the central region from compressing the dynamic range used for thresholding, a min–max rescaling is used whose extrema are computed over Ω only. Define $X_{\min}^{\Omega }=\min_{(i,j)\in \Omega }X\left(i,j\right)$ and $X_{\max}^{\Omega }=\max_{(i,j)\in \Omega }X\left(i,j\right)$. The operator $R_{\Omega }\left(X\right)$ therefore performs min–max normalization using extrema computed over pixels in Ω only, which prevents values outside Ω from compressing contrast in the region used for threshold selection. The stabilizer ϵ ensures numerical safety when $\max_{\Omega }X\approx \min_{\Omega }X$.

(14) $$R_{\Omega }\left(X\right)\left(u,v\right)=\left\{\begin{array}{cc} \frac{X\left(u,v\right)-X_{\min}^{\Omega }}{X_{\max}^{\Omega }-X_{\min}^{\Omega }+\epsilon }, & (u,v)\in \Omega ,\\ 0, & (u,v)\notin \Omega . \end{array}\right.$$

A local SSIM map is computed between $\tilde{I}$ and $T_{s}$. For a window centered at (u,v), let $\mu_{\tilde{I}},\mu_{T}$ be local means, let $\sigma_{\tilde{I}}^{2},\sigma_{T}^{2}$ be local variances, and let $\sigma_{\tilde{I}T}$ be the local covariance. The SSIM map value can be written as

(15) $$\begin{array}{c} S\left(u,v\right)=\frac{\left(2\mu_{\tilde{I}}\mu_{T}+C_{1}\right)\left(2\sigma_{\tilde{I}T}+C_{2}\right)}{\left(\mu_{\tilde{I}}^{2}+\mu_{T}^{2}+C_{1}\right)\left(\sigma_{\tilde{I}}^{2}+\sigma_{T}^{2}+C_{2}\right)}, \end{array}$$

where $C_{1},C_{2}$ stabilize the ratio. This is converted to a structural dissimilarity map,

(16) $$D_{\mathrm{ssim}}\left(u,v\right)=1-S\left(u,v\right).$$

In our implementation, the SSIM map is computed using MATLAB R2023a’s SSIM function with explicitly specified parameters (window size/weighting and stabilizing constants) for reproducibility; the exact values are reported in Appendix A.4. An absolute-difference map is also computed

(17) $$D_{\mathrm{abs}}\left(u,v\right)=\left|\tilde{I}\left(u,v\right)-T_{s}\left(u,v\right)\right|.$$

An additive fusion with weight *w* is used, followed by rescaling for stable thresholding:

(18) $$\begin{array}{cc} M_{\mathrm{raw}}\left(u,v\right) & =D_{\mathrm{ssim}}\left(u,v\right)+w\;R_{\Omega }\left(D_{\mathrm{abs}}\right)\left(u,v\right), \end{array}$$

(19) $$\begin{array}{cc} M(u,v) & =R_{\Omega }\left(M_{\mathrm{raw}}\left(u,v\right)\right). \end{array}$$

In this interpretation, M(u,v) is a defect-likelihood signal: localized defects produce concentrated peaks, while nuisance global mismatch (wrong template) tends to produce broad responses.

### 2.4. Mask and Box Extraction

Defects are sparse, so a high-quantile threshold is chosen using pixels in Ω only:

(20) $$\theta =Q_{q}\left(\{M(u,v):(u,v)\in \Omega \}\right),$$

where q∈(0,1) is a sensitivity parameter.

For a finite set of values ${\left\{z_{i}\right\}}_{i=1}^{n}$, the *q*-quantile $Q_{q}$ is any value such that at least a fraction *q* of samples are $\leq Q_{q}$ and at least a fraction (1−q) are $\geq Q_{q}$. Using a high quantile to set θ is robust in this setting because true defects typically occupy only a small fraction of pixels within Ω.

The initial binary mask is

(21) $$Y_{0}\left(u,v\right)=⊮\left[M\left(u,v\right)>\theta \right]\;⊮\left[\left(u,v\right)\in \Omega \right].$$

To suppress speckle noise and consolidate defect blobs, a sequence of set operators is applied to $Y_{0}$ within the inner region. Let B(r) denote a disk structuring element of radius *r*, and let $\mathrm{AreaOpen}(\cdot ;A_{\min})$ remove connected components smaller than $A_{\min}$. The sequence is

(22) $$\begin{array}{cc} Y_{1} & =\mathrm{AreaOpen}(Y_{0};A_{\min}), \end{array}$$

(23) $$\begin{array}{cc} Y_{2} & =Y_{1}•B\left(r_{c}\right), \end{array}$$

(24) $$\begin{array}{cc} Y_{3} & =Y_{2}⊕B\left(r_{d}\right), \end{array}$$

(25) $$\begin{array}{cc} Y & =Y_{3}\wedge {⊮}_{\Omega }, \end{array}$$

where • is morphological closing, ⊕ is dilation, and ∧ is pixel-wise AND with the inner-region mask ${⊮}_{\Omega }$.

Connected components of *Y* are converted to axis-aligned bounding boxes b=[xywh]. Each component is assigned a confidence score using a robust statistic of *M* inside the component. A stable choice is a high quantile:

(26) $$s_{i}=Q_{q_{s}}\left(\{M\left(u,v\right):\left(u,v\right)\in C_{i}\}\right),\;q_{s}\in \left(0,1\right).$$

Boxes are sorted by $s_{i}$ and mapped back to the original resolution if the processing scale s<1. For the exact connected-component scoring rule and supported alternatives (e.g., quantile/max/mean scoring), see Appendix A.5. Therefore, the final output of this stage is a confidence-ranked set of detections

(27) $$P={\left\{\left(b_{i},s_{i}\right)\right\}}_{i=1}^{N},$$

where each $b_{i}=\left[x\;y\;w\;h\right]$ is an axis-aligned bounding box, and $s_{i}$ is its confidence score derived from the defect-likelihood field. In summary, the mask-and-box stage is controlled by a small set of interpretable parameters: threshold quantile *q*, minimum area $A_{\min}$, closing radius $r_{c}$, dilation radius $r_{d}$, and confidence quantile $q_{s}$ (default $q_{s}=0.95$). These parameters jointly control the false-positive/false-negative trade-off and the coarseness of the resulting boxes.

### 2.5. Algorithm Summary

**Figure 2:**
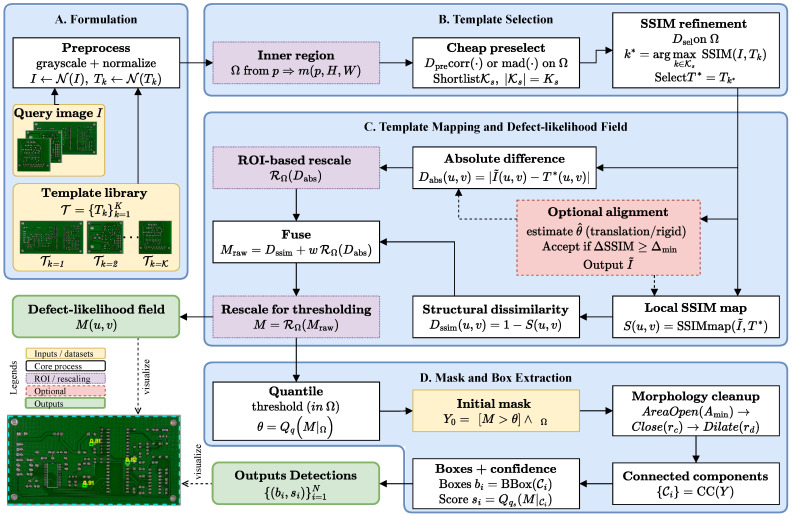
Mathematical overview of the proposed image processing framework.

The proposed framework takes a defective query image and selects a reference template from a small library using a coarse-to-fine strategy (cheap pre-screening followed by SSIM refinement). If enabled, a global alignment is applied with an explicit acceptance test based on SSIM gain on the inner region, limiting transforms biased by defect content. A defect-likelihood field is then constructed by fusing an SSIM-derived structural dissimilarity map with a normalized absolute-difference map and applying an Ω-based rescaling. Finally, high-quantile thresholding and simple morphological post-processing yield connected components that are converted into confidence-ranked bounding boxes. Algorithm 1 summarizes the full procedure, while the mathematical operators and data products are summarized in Figure 2.
**Algorithm 1** Template selection and defect localization**Require:** Query image *I*, templates ${\left\{T_{k}\right\}}_{k=1}^{K}$,     parameters $\left(p,w,q,q_{s},A_{\min},r_{c},r_{d},D_{\mathrm{pre}},D_{\mathrm{sel}},D_{\mathrm{proc}},K_{s},\Delta_{\min}\right)$**Ensure:** Detections $P=\{\left(b_{i},s_{i}\right)\}$ and defect-likelihood field *M*  1:Compute central region Ω from area-avoidance ratio *p*  2:Preselect shortlist $K_{s}$ (size $K_{s}$) using a cheap score at resolution $D_{\mathrm{pre}}$  3:Select template $T^{*}$ by maximizing SSIM on Ω over $k\in K_{s}$ at resolution $D_{\mathrm{sel}}$  4:Set processing scale *s* from $D_{\mathrm{proc}}$ and build processed images $\left(I_{s},T_{s}\right)$  5:Optional alignment: estimate transform $\hat{\theta }$ and compute SSIM gain Δ on Ω; accept if $\Delta \geq \Delta_{\min}$  6:Define aligned query $\tilde{I}$ (either warped $I_{s}$ or $I_{s}$)  7:Compute SSIM map S(u,v) between $\tilde{I}$ and $T_{s}$  8:Compute dissimilarity maps $D_{\mathrm{ssim}}\left(u,v\right)=1-S\left(u,v\right)$ and $D_{\mathrm{abs}}\left(u,v\right)=\left|\tilde{I}\left(u,v\right)-T_{s}\left(u,v\right)\right|$  9:Fuse and rescale to obtain defect-likelihood field *M*10:Threshold: $\theta =Q_{q}\left(\left\{M\left(u,v\right):\left(u,v\right)\in \Omega \right\}\right)$11:Initial mask: $Y_{0}=⊮\left[M>\theta \right]\wedge {⊮}_{\Omega }$12:Cleanup: $Y=\mathrm{AreaOpen}\left(Y_{0};A_{\min}\right)•B\left(r_{c}\right)⊕B\left(r_{d}\right)$13:Connected components $\left\{C_{i}\right\}$→ boxes $\left\{b_{i}\right\}$ and scores $s_{i}=Q_{q_{s}}\left(\left\{M\left(u,v\right):\left(u,v\right)\in C_{i}\right\}\right)$14:Map boxes back to original scale (if s<1)

#### Computational Complexity

Let *K* be the number of available templates and $K_{s}\ll K$ be the shortlist size after preselection. Let $D_{\mathrm{pre}},D_{\mathrm{sel}},D_{\mathrm{proc}}$ denote the maximum long-side resolutions used for (i) the cheap preselect score, (ii) SSIM refinement for template selection, and (iii) the downstream processing grid, respectively. Using the common square-grid proxy, the number of evaluated pixels scales as $O(D^{2})$ at resolution *D*. Restricting computations to the central region Ω (via ROI cropping/masking) primarily changes constants. Per query image, the coarse-to-fine template selection cost is

(28) $$O\left(K\;D_{\mathrm{pre}}^{2}+K_{s}\;D_{\mathrm{sel}}^{2}\right),$$

where the first term corresponds to evaluating a cheap preselect score over all templates at $D_{\mathrm{pre}}$, and the second term corresponds to SSIM refinement over the shortlist at $D_{\mathrm{sel}}$ (dominant when enabled). After selecting $T^{*}$, the downstream stages operate on the processing grid at $D_{\mathrm{proc}}$: (i) optional global alignment uses a correlation-based registration objective (translation/rigid) and in practice scales near-linearly in the number of processed pixels (often with FFT-accelerated correlation), (ii) the SSIM map uses a fixed window size (constant window operations per pixel), and (iii) difference-map construction, Ω-based rescaling, quantile thresholding, morphology with fixed structuring-element radii, and connected-components labeling are all $O(D_{\mathrm{proc}}^{2})$ up to small multiplicative constants. Overall, the per-image runtime scales as

(29) $$O\left(K\;D_{\mathrm{pre}}^{2}+K_{s}\;D_{\mathrm{sel}}^{2}+D_{\mathrm{proc}}^{2}\right),$$

and the dataset runtime is linear in the number of evaluated images. In practice, template preprocessing (grayscale/normalization/resizing) can be cached, and choosing $D_{\mathrm{pre}}\ll D_{\mathrm{sel}}$ with $K_{s}\ll K$ keeps selection tractable even for moderate template libraries.

### 2.6. Experimental Setup and Evaluation Protocol

Experiments use the PKU-Market-PCB dataset [3]. Defect classes are used only for class-wise reporting; the localization pipeline itself is class-agnostic across defect categories (while assuming a valid defect-free reference template for the same layout/revision). The dataset folder organization (templates, defective images, and Pascal Visual Object Classes (VOC) annotations) is described in Appendix A.6. The proposed method is implemented in MATLAB R2023a, and experiments are run on an Apple M1 processor with 8GB RAM. The implementation configuration and hyperparameters are reported in Appendix A.7

#### 2.6.1. Dataset

Although the proposed method employs training-free image processing that produces an explicit defect-likelihood field M(u,v) and interpretable intermediate maps, the final outputs are evaluated using standard box-level localization metrics, which facilitates comparison with established benchmarks in the AOI and object-detection literature. For broader context on automated optical inspection in electronics manufacturing and recent perspectives on PCB defect detection, see the comprehensive surveys in [69,70]. Table 1 summarizes the evaluated dataset, including image resolution and annotation statistics.

#### 2.6.2. Detections and Confidence

For each query image, the method outputs a set of axis-aligned detections $P={\left\{\left(b_{i},s_{i}\right)\right\}}_{i=1}^{N_{P}}$, where each $b_{i}=\left[x\;y\;w\;h\right]$ is the bounding box of a connected component extracted from a thresholded version of M(u,v) (Section 2.4). Each detection is assigned a confidence score $s_{i}$ computed as a robust statistic of *M* values inside the component (a high-quantile score is used so that a higher $s_{i}$ indicates stronger evidence of a localized peak). For the exact component-to-box confidence definition used in the implementation, see Appendix A.5.

#### 2.6.3. Ground Truth and Intersection-over-Union (IoU) Matching

Ground-truth annotations are axis-aligned Pascal VOC boxes. For each query image, let $G={\left\{g_{j}\right\}}_{j=1}^{N_{G}}$ denote the set of ground-truth boxes. Detections are matched to ground truth using a greedy one-to-one assignment within each image: detections are processed in descending confidence order, and a detection $b_{i}$ is matched to an as-yet-unmatched $g_{j}$ if $\mathrm{IoU}\left(b_{i},g_{j}\right)\geq \tau_{\mathrm{IoU}}$ (each detection matches at most one ground-truth box and vice versa). Unmatched detections are counted as false positives, and unmatched ground-truth boxes are counted as false negatives.

Unless noted otherwise, $\tau_{\mathrm{IoU}}=0.10$ is used because detections are derived from thresholded connected components rather than tight box regression.

#### 2.6.4. Fixed Operating Point (Counts and F1)

Given a score threshold *t*, detections with $s_{i}\geq t$ are retained and dataset-level counts (after per-image one-to-one matching) are computed: true positives TP(t), false positives FP(t), and false negatives FN(t). Precision, recall, and F1 are

(30) $$\begin{array}{cc} \mathrm{Precision}(t) & =\frac{\mathrm{TP}(t)}{\mathrm{TP}(t)+\mathrm{FP}(t)+\epsilon }, \end{array}$$

(31) $$\begin{array}{cc} \mathrm{Recall}(t) & =\frac{\mathrm{TP}(t)}{\mathrm{TP}(t)+\mathrm{FN}(t)+\epsilon }, \end{array}$$

(32) $$\begin{array}{cc} F1(t) & =\frac{2\;\mathrm{Precision}(t)\;\mathrm{Recall}(t)}{\mathrm{Precision}(t)+\mathrm{Recall}(t)+\epsilon }. \end{array}$$

where ϵ>0 is a small numerical stabilizer (e.g., $10^{-12}$) used only to avoid division-by-zero in degenerate cases (e.g., when TP(t)+FP(t)=0); it does not affect results when denominators are nonzero.

For completeness, we also report a detection accuracy that does not require defining true negatives (TN), which are not well-defined for variable-length detection outputs:

(33) $$\mathrm{Accuracy}\left(t\right)=\frac{\mathrm{TP}(t)}{\mathrm{TP}(t)+\mathrm{FP}(t)+\mathrm{FN}(t)+\epsilon }.$$

#### 2.6.5. Avoiding Threshold Selection on the Reported Set

To avoid optimistic bias from selecting *t* on the same set used for reporting, operating-point metrics are reported using *K*-fold cross-validation: in each fold, the score threshold $t^{*}$ is chosen to maximize F1(t) on the calibration split (the union of the other K−1 folds), and precision/recall/F1 are then computed on the held-out fold using this fixed threshold. This work reports the mean ± standard deviation across folds. For continuity with the original submission and to aid interpretation of class-wise behavior, this also includes a reference operating point obtained by selecting $t^{*}$ on the full evaluated set; this reference is explicitly labeled and is not used as the primary estimate of generalization performance. Implementation details for the cross-validated operating point selection are provided in Appendix B.1.

#### 2.6.6. Threshold-Sweep Metrics (PR/AP, AP-Versus-IoU, and FROC)

To summarize performance across confidence thresholds, the precision–recall curve is constructed by ranking all detections across the dataset by confidence $s_{i}$ and sweeping down this global ranked list. True/false positives are determined using the matching protocol described above at the specified $\tau_{\mathrm{IoU}}$ [71,72]. Average precision (AP) is computed from this PR curve using a precision envelope and piecewise integration over recall (the VOC07 11-point approximation is disabled in the evaluation script).

To quantify sensitivity to localization stringency, AP is also computed across multiple IoU thresholds. In particular, (i) a custom sweep $\tau_{\mathrm{IoU}}\in \left\{0.10,0.20,\ldots ,0.90\right\}$ and (ii) a COCO-style sweep $\tau_{\mathrm{IoU}}\in \left\{0.50,0.55,\ldots ,0.95\right\}$ are reported. When multiple IoU thresholds are considered, mean Average Precision mAP is reported as the mean AP across the specified thresholds.

For operational analysis, free-response receiver operating characteristic (FROC) curves are reported by sweeping score thresholds. For a threshold *t*, let $N_{\mathrm{img}}$ be the number of evaluated images, let $N_{\mathrm{GT}}$ be the total number of ground-truth boxes, and let $N_{\mathrm{img}}^{\mathrm{GT}}$ be the number of images containing at least one ground-truth box. The following quantities are defined:

(34) $$\begin{array}{cc} \mathrm{FP}/\mathrm{image}(t) & =\frac{\mathrm{FP}(t)}{N_{\mathrm{img}}+\epsilon }, \end{array}$$

(35) $$\begin{array}{cc} {\mathrm{Recall}}_{\mathrm{obj}}\left(t\right) & =\frac{\mathrm{TP}(t)}{N_{\mathrm{GT}}+\epsilon }, \end{array}$$

(36) $$\begin{array}{cc} {\mathrm{Recall}}_{\mathrm{img}}\left(t\right) & =\frac{N_{\mathrm{img}}^{\mathrm{TP}}\left(t\right)}{N_{\mathrm{img}}^{\mathrm{GT}}+\epsilon }, \end{array}$$

where $N_{\mathrm{img}}^{\mathrm{TP}}\left(t\right)$ is the number of ground-truth-containing images for which at least one detection is a matched true positive (TP) at threshold *t*. In this paper, FROC plots and operating-point tables use object-level recall ${\mathrm{Recall}}_{\mathrm{obj}}\left(t\right)$ versus FP/image(t); image-level recall is included for completeness. The exported threshold grid and image-level recall computation used to generate the FROC bundle are described in Appendix B.2.

At the dataset level, $N_{\mathrm{GT}}=\mathrm{TP}\left(t\right)+\mathrm{FN}\left(t\right)$; therefore, Recall(t) and ${\mathrm{Recall}}_{\mathrm{obj}}\left(t\right)$ are numerically equivalent. Accordingly, ${\mathrm{Recall}}_{\mathrm{obj}}\left(t\right)$ is used in the FROC definition to emphasize the object-level nature of the curve.

For completeness, explicit definitions for IoU on [xywh] boxes and the AP computation used in our evaluation are provided in Appendices Appendix B.3 and Appendix B.4.

## 3. Experimental Results and Analysis

This section reports qualitative examples and quantitative localization performance under the experimental setup and evaluation protocol described in the previous subsection. The method produces confidence-ranked detections $P=\{\left(b_{i},s_{i}\right)\}$ from an explicit defect-likelihood field M(u,v); therefore, results are presented both at a fixed operating point (counts and precision/recall/F1) and across confidence thresholds (precision–recall, AP, and FROC).

All quantitative results in this section are reported on 693 evaluated query images under the adopted IoU matching protocol (Section 2.6). Threshold-sweep curves (PR/AP/FROC) are computed on the full set. For fixed-threshold operating-point metrics, this work reports *K*-fold cross-validated results (mean ± std) to avoid selecting thresholds on the same set used for reporting.

For reference and continuity with prior results, the manuscript also reports descriptive counts at a reference best-F1 threshold selected on the full evaluated set (explicitly labeled as reference).

### 3.1. Qualitative Results and Failure Modes

**Figure 3:**
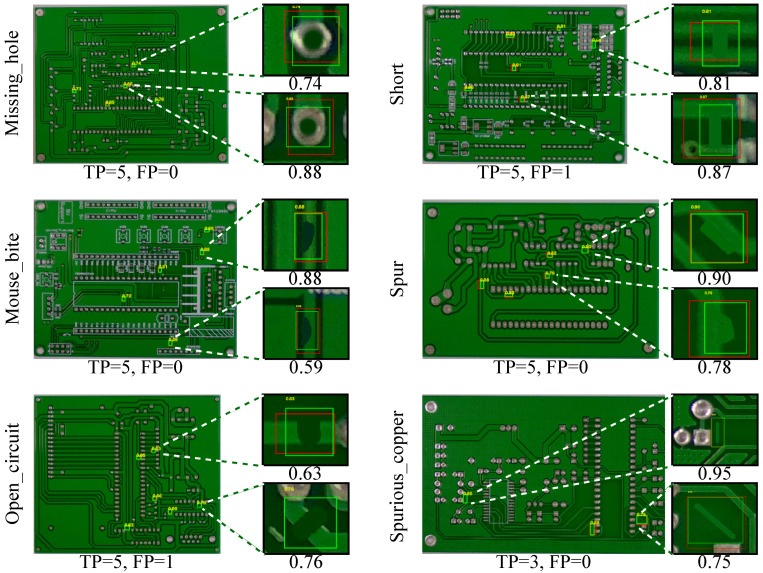
Example true positive cases (red: ground truth; green: proposed detection).

Figure 3 shows representative TP cases where the proposed mapping produces localized peaks in M(u,v) that translate into axis-aligned bounding boxes overlapping the annotated defects. While the boxes are not optimized to tightly fit the defect boundary (they are derived from thresholded connected components), they consistently localize the defect region and provide a monotonic confidence score $s_{i}$ for ranking.

**Figure 4:**
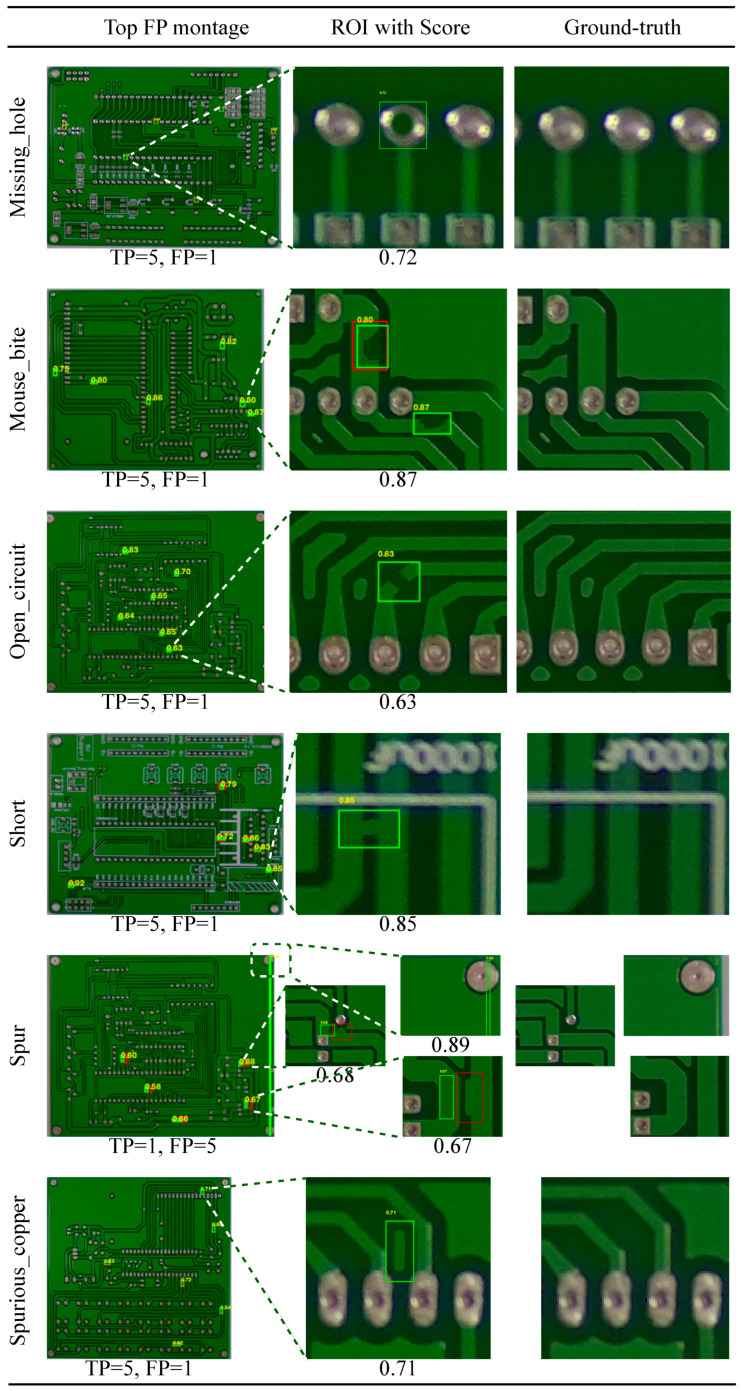
Example false positive cases (red: ground truth; green: proposed detection).

Figure 4 shows representative false positive (FP) cases. In practice, FPs tend to arise from nuisance variability that produces structured responses in the SSIM/difference maps, such as residual global misalignment, illumination drift, or local texture/reflectance changes that are not labeled as defects. A qualitative review of these cases suggests that many FPs stem from two primary sources: (i) structured nuisance variability such as residual global misalignment, illumination drift [73], or local texture changes that are not true defects, and (ii) actual defects that are present in the images but are missing from the corresponding Pascal VOC XML annotation files. Because this work operates strictly under the provided ground-truth labels without resorting to relabeling or manual verification, all reported metrics faithfully reflect the adopted box-matching protocol and evaluation set. The presence of unlabeled defects in the FP set underscores the importance of careful annotation in benchmark datasets and suggests that true recall may be higher than reported. These observations underscore the value of the optional alignment safeguard and careful template selection, which are most influential in reducing nuisance-driven artifacts. Moreover, the confidence-ranked outputs and FROC curves (Section 3.6) enable operational threshold selection based on a tolerable false-alarm budget in production, allowing practitioners to adjust operating points according to their acceptable FP/image rate and trade recall for lower false-alarm burden when necessary.

**Figure 5:**
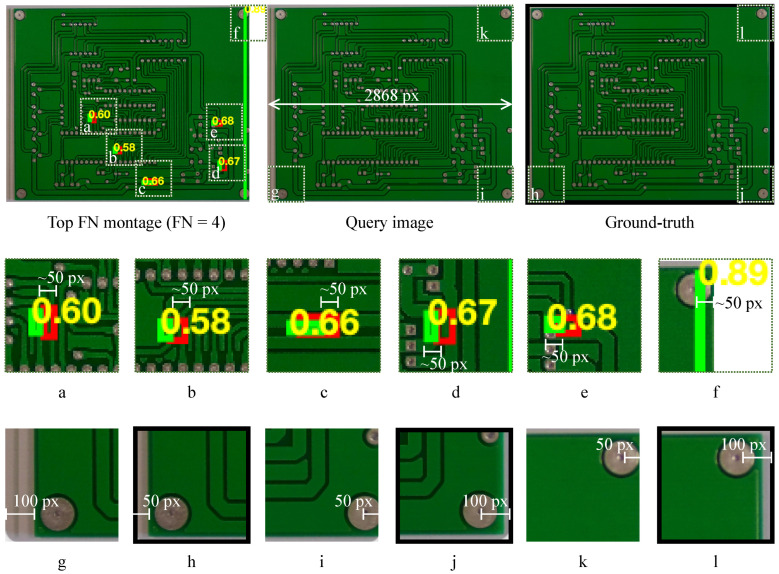
Example false negative case (red: ground truth; green: proposed detection; (**a**–**f**) are corresponding crop regions of the Top FN montage; (**g**,**i**,**k**) are crop regions of the query image; (**h**,**j**,**l**) are crop regions of the reference ground-truth image).

Figure 5 provides representative visual evidence for the observed Spur false negatives (all four FNs in the per-class breakdown). These cases are consistent with thin/fragmented structures producing a weaker or spatially discontinuous response under the current high-quantile thresholding and morphology settings, which can suppress small components or yield boxes that fail the one-to-one IoU matching criterion. Because the pipeline is deterministic and controlled by interpretable parameters $\left(q,A_{\min},r_{c},r_{d},q_{s}\right)$, this behavior reflects an explicit operating trade-off that can be adjusted (as also summarized by the FROC curve in Section 3.6).

### 3.2. Template Selection Quality and Practical Failure Causes

The exported per-image diagnostics show that template selection is typically highly stable (template SSIM values are near 1.0 for the majority of images), but rare outliers can occur, which are consistent with occasional template mismatch or nontrivial capture variation. On the 693 evaluated images, the per-image template SSIM has median ≈0.9991 (p5 ≈0.9976), while a small number of outliers fall below 0.99 (4/693 images) and one extreme case reaches as low as ≈0.4967. These outliers are practically important because low similarity can inflate structured responses in M(u,v) and therefore increase false positives under fixed post-processing parameters.

### 3.3. Quantitative Summary at the Adopted Operating Point

Table 2 reports per-class detection counts (TP/FP/FN) and the resulting precision/recall/F1 under the adopted IoU matching protocol at a reference best-F1 operating point selected on the full evaluated set. Overall, the method achieves TP = 2949, FP = 103, FN = 4, yielding Precision = 0.9663, Recall = 0.9987, and F1 = 0.9822 at this reference threshold. Cross-validated operating-point results (mean ± std across folds), which avoid threshold selection on the reported set, are provided in Table 3. In this revision, we treat these cross-validated operating-point results as the primary generalization-oriented summary, while the full-set best-F1 operating point is retained only as a clearly labeled reference point.

Across defect types, performance is broadly consistent. The main deviations are informative: all four missed defects (FN = 4) occur in the Spur class, suggesting a small subset of cases where the defect response is weaker or more fragmented under the current post-processing parameters. This behavior is expected in a deterministic pipeline and can be addressed by adjusting the interpretable parameters $\left(q,A_{\min},r_{c},r_{d},q_{s}\right)$ for the desired false-positive/false-negative trade-off.

### 3.4. Precision–Recall Characteristics Across Confidence Thresholds

**Figure 6:**
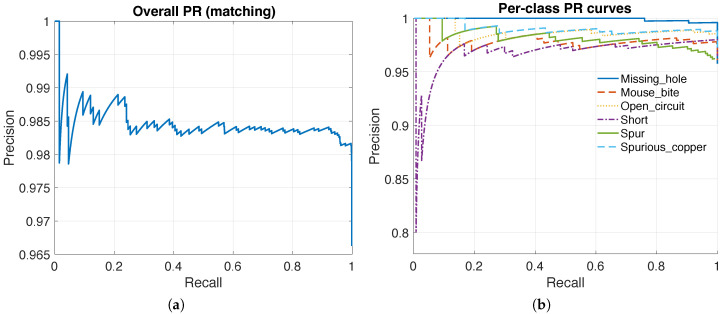
Precision–recall curves under the IoU matching protocol. (**a**) Overall PR curve. (**b**) Per-class PR curves.

Because detections are confidence-ranked by $s_{i}$, varying the acceptance threshold induces a precision–recall (PR) trade-off. Figure 6 reports the overall PR curve and the per-class PR curves, summarizing how precision changes as recall increases and which defect types degrade earlier as the threshold is lowered.

In AOI practice, this view is useful because the operating point is chosen based on the acceptable false-alarm burden in production. In addition, the PR curves validate that the proposed confidence score is informative: a meaningful ranking yields a PR curve that maintains high precision over a wide recall range.

### 3.5. Sensitivity to Matching Stringency (AP Versus IoU)

**Figure 7:**
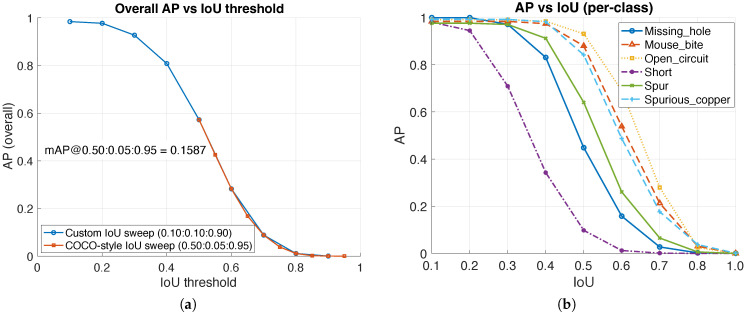
AP versus IoU threshold. (**a**) Overall AP versus IoU threshold, including custom IoU sweep and optional COCO-style mAP summary. (**b**) Per-class AP versus IoU threshold.

Figure 7 reports average precision as a function of the IoU threshold. As the IoU requirement becomes stricter, AP decreases because the proposed detections are derived from thresholded map components and are not explicitly optimized to tightly fit the ground-truth boxes. In particular, because our boxes are obtained by extracting connected components and enclosing them with axis-aligned rectangles, $\tau_{\mathrm{IoU}}=0.10$ should be interpreted as a permissive matching criterion for region-level localization (component fragmentation/merging can yield looser boxes even when the detected region overlaps the defect). Consequently, the decline in AP at higher IoU thresholds primarily reflects box tightness/placement mismatch rather than an inability to detect defect regions. Quantitatively, the exported AP–IoU sweep shows high AP at the adopted low-stringency regime (overall AP ≈ 0.984 at IoU = 0.10) but a pronounced decline at stricter thresholds (overall AP ≈ 0.572 at IoU = 0.50), approaching near-zero at very strict IoU (e.g., ≥0.80). This plot is therefore included to transparently characterize localization-stringency sensitivity rather than to claim pixel-tight bounding-box regression.

For completeness, Table 4 first summarizes representative overall AP values at selected IoU thresholds, and Figure 7 then visualizes the full AP-versus-IoU behavior, including both the overall curves (custom and COCO-style sweeps) and the per-class curves.

### 3.6. False-Alarm Rate Versus Recall (Or FROC)

**Figure 8:**
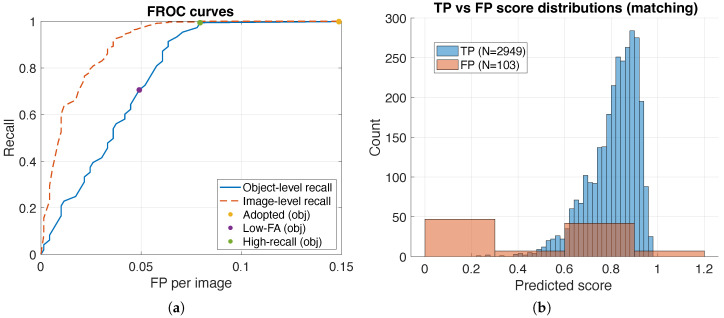
Operational views for threshold selection. (**a**) Free-response receiver operating characteristic FROC curves. (**b**) True positive and false positive score distributions histograms.

For deployment, a key question is how many false alarms must be tolerated to achieve a target recall. Figure 8 reports FROC curves, which summarize recall as a function of the false positive rate (e.g., false positives per image) as the confidence threshold varies. This view complements PR curves by emphasizing the operational cost of additional recall.

At the adopted operating point (Table 2), the system attains Recall = 0.9987 with FP/image ≈ 0.149. In practice, Figure 8 is used to choose an operating threshold based on an allowed FP/image budget; the exported FROC curve enables selecting alternative thresholds to trade a small recall reduction for a lower false-alarm burden.

### 3.7. Score Separability of True and False Detections

Figure 8 compares the confidence-score distributions of true positives and false positives. A clear separation indicates that the confidence score $s_{i}$ provides a meaningful ranking signal, enabling threshold selection to trade precision for recall. Overlap between TP and FP score distributions highlights ambiguous cases (e.g., residual misalignment or illumination-driven artifacts) that can be addressed by improving template selection, enabling alignment when appropriate, or adjusting the mask-and-box parameters.

### 3.8. Quantitative Runtime Breakdown

Beyond asymptotic scaling, an empirical runtime breakdown of the reference implementation by stage is reported in Table 5.

## 4. Discussion

The proposed framework targets practical AOI where defect-free references are available and decisions must be reliable, auditable, and deployable without a training lifecycle. Operating-point metrics are summarized using *K*-fold cross-validation to avoid selecting thresholds on the reported set (Table 3), while a reference best-F1 operating point on the full set is retained for descriptive continuity (Table 2). Under the adopted IoU matching protocol, confidence-ranked outputs support operational interpretation via PR/FROC sweeps (Figure 6 and Figure 8) and IoU-sensitivity via AP-versus-IoU analysis (Figure 7).

### 4.1. Why the Method Works in Practice

Robustness comes from explicitly controlling nuisance variability and using structure-aware comparison: template selection reduces systematic mismatch, optional alignment is gated by SSIM gain to avoid defect-driven over-correction, and the fused defect-likelihood field combines SSIM-derived structural dissimilarity with normalized absolute difference to handle both structural and radiometric changes (e.g., illumination drift). Together, these choices yield a confidence score that is informative for ranking, consistent with the PR and FROC behavior.

### 4.2. Class-Wise Behavior and Failure Modes

Per-class performance is broadly consistent; the main deviation is that the only missed defects (FN = 4) occur in the Spur class (Table 2), where responses can be weaker or more fragmented under the current post-processing. This is expected in a deterministic connected-components-to-box stage (threshold/area/morphology can suppress fine structures), and recall can be increased by adjusting $\left(q,A_{\min},r_{c},r_{d},q_{s}\right)$ with the resulting FP/image quantified by the FROC curve.

### 4.3. Operating-Point Selection and Localization Stringency

Operating-point metrics are reported using *K*-fold cross-validation to avoid selecting thresholds on the same set used for reporting (Section 2.6). In practice, deployment typically selects thresholds based on an allowable false-alarm budget; Figure 8 and Table 6 make this trade-off explicit. Accordingly, FROC (object-level recall versus FP/image) is recommended as the primary operational view, with PR as a complementary precision summary. The adopted matching threshold $\tau_{\mathrm{IoU}}=0.10$ reflects that detections are derived from thresholded connected components rather than tight box regression; consequently, AP decreases as IoU becomes stricter (Figure 7), indicating a design choice favoring reliable region localization for QC over pixel-tight boxes. To avoid over-interpreting performance under low-stringency matching, the Results section summarizes AP at representative moderate IoU thresholds and provides the full AP-versus-IoU sweep. If tighter boxes are required, a lightweight post-processing refinement (e.g., contour-based tightening) can be added without changing the detection ranking.

### 4.4. Limitations and Practical Considerations

The method assumes defect-free templates of the same PCB layout/revision, which is realistic in many manufacturing settings and helps resolve defect-versus-intent ambiguity; however, this assumption also defines the primary limitations:

- Layout/revision mismatch or wrong template: if the selected template does not correspond to the same board revision (or if no valid template exists), reference comparison can yield structured false-positive regions that mimic defects. Coarse-to-fine template selection mitigates mild mismatch, but it cannot correct true design changes; in such cases, the correct remedy is updating the template library (or enforcing recipe-based template selection in fixed-product lines).
- Perspective/projective distortion and nonrigid deformation: the SSIM-gated alignment improves robustness to small global shifts/rotations, but larger viewpoint/perspective changes remain out of scope. When the imaging geometry is not fixture-controlled, residual misregistration can dominate difference/SSIM maps and reduce localization quality.
- Heavy illumination non-uniformity and reflectance drift: normalization and the fused SSIM/difference design reduce sensitivity to global brightness drift; however, strong spatially varying illumination (e.g., vignetting, hotspots, and specular highlights) can still introduce structured responses in the defect-likelihood field and increase false positives.
- ROI-based statistics and border effects: evaluating thresholds and similarity on the central region Ω improves robustness to border artifacts, but defects near the excluded border can have reduced influence on thresholding statistics. If border defects are critical, the ROI margin should be reduced (with the expected trade-off of higher nuisance sensitivity).
- Component-derived boxes are not tight boundaries: detections are produced from thresholded connected components, so bounding boxes are intended for reliable region localization rather than pixel-tight boundaries. This is why AP drops at stricter IoU thresholds (Figure 7); if tighter boxes are required, a lightweight contour-based tightening step can be added without changing the detection ranking.

Finally, this revision does not include trained deep learning baselines; because training-based results can be sensitive to split definition and training/engineering choices, we treat such comparisons as out of scope here and instead report explicit PR/AP/FROC and IoU-sensitivity analyses under a fully specified protocol.

### 4.5. Future Work

Future work includes the following: (i) automatic threshold recommendation for a specified FP/image budget from the exported FROC curve; (ii) lightweight box tightening to improve IoU without retraining; (iii) broader alignment models (e.g., rigid/affine) while retaining an SSIM-gain acceptance gate; (iv) per-template caching and template-library management for scale; and (v) computational/implementation optimizations (early exits, efficient SSIM/difference computation, downsampling-aware parameter scaling, and fixed-point/embedded-friendly variants) with evaluation on single-board computers or microcontrollers.

## 5. Conclusions

This work addresses defect–intent ambiguity in PCB AOI by presenting a training-free, reference-based defect localization framework designed for deployments where interpretability and controllable false-alarm rates are critical. The method selects a compatible defect-free reference via coarse-to-fine matching, applies global alignment only when it increases SSIM (to avoid defect-driven over-correction), and forms a defect-likelihood field by fusing SSIM-derived structural dissimilarity with normalized absolute differences; confidence-ranked detections are then produced via connected-component extraction.

To support deployment-oriented decision making, we report precision–recall and FROC curves rather than advocating a single universal threshold. At the adopted operating point, the method achieves 0.9987 recall at 0.149 FP/image, and operating-point metrics are additionally reported using *K*-fold calibration/test thresholding to avoid selecting thresholds on the same set used for reporting (with a full-set best-F1 threshold retained only as a clearly labeled reference point). Under the adopted box-matching protocol with $\tau_{\mathrm{IoU}}=0.10$, average precision reaches 0.984, and AP-versus-IoU sweeps transparently characterize sensitivity to stricter localization requirements. The primary limitation is the need for defect-free templates of the same PCB layout/revision; within this setting, the approach provides a reproducible and auditable alternative to training-based inspection by flagging only statistically and structurally supported deviations for review.

## Figures and Tables

**Table 1:** Dataset summary.

Item	Value
Defective images (run)	693
Evaluated images (with GT XML)	693
Defect-free templates	10
Defect classes	6
GT objects (total)	2953
Median resolution (W × H in pixels)	2868 × 2159
Resolution range (W × H in pixels)	2240–3056 × 1586–2530

**Table 2:** Reference operating-point detection counts and metrics (threshold selected on the full evaluated set).

Class	TP	FP	FN	Prec.	Rec.	F1	Acc.
Missing_hole	497	22	0	0.9576	1.0000	0.9784	0.9576
Mouse_bite	492	20	0	0.9609	1.0000	0.9801	0.9609
Open_circuit	482	8	0	0.9837	1.0000	0.9918	0.9837
Short	491	18	0	0.9646	1.0000	0.9820	0.9646
Spur	484	19	4	0.9622	0.9918	0.9768	0.9546
Spurious_copper	503	16	0	0.9692	1.0000	0.9843	0.9692
**Overall**	2949	103	4	0.9663	0.9987	0.9822	0.9650

**Table 3:** Cross-validated operating-point metrics (mean ± std across folds).

Metric	Mean ± Std	Notes
Threshold	0.322 ± 0.053	K = 5, IoU = 0.10
Precision	0.980 ± 0.008	
Recall	0.997 ± 0.003	
F1	0.988 ± 0.004	
FP per image	0.088 ± 0.035	

**Table 4:** AP at representative IoU thresholds (overall).

AP	0.05	0.10	0.20	0.30	0.40	0.50
IoU	0.985	0.984	0.977	0.927	0.808	0.572

**Table 5:** Runtime breakdown by stage (reference implementation).

Stage	Time (s/Image)
Template selection (total)	0.242
Preselect	0.109
SSIM refine	0.033
Localization (total)	0.793
Preprocess	0.043
Resize	0.059
Align	0.368
Score map	0.048
Mask	0.073
Boxes	0.004
Total	1.035

**Table 6:** Example operating points derived from the exported FROC curve (object-level recall versus FP/image).

Operating Point	FP/Image	Recall	Notes
Adopted threshold	0.149	0.9987	From Table 2
Low-false-alarm	0.049	0.7057	scoreThr = 0.7712 (max recall with FP/image ≤ 0.05)
High-recall	0.079	0.9939	scoreThr = 0.4441 (min FP/image with recall ≥ 0.99)

## Data Availability

The code supporting the findings of this study will be finalized and released publicly upon acceptance/publication at: https://github.com/nonsakhoo/PCB-defect-localization (accessed on 24 January 2026). During peer review, an unofficial snapshot is made available for reviewers via the same repository URL.

## Abbreviations

    The following abbreviations are used in this manuscript:

Acc.	Accuracy
AI	Artificial Intelligence
AOI	Automated Optical Inspection
AP	Average Precision
EMC	Electromagnetic Compatibility
EMI	Electromagnetic Interference
F1	F1 Score
FN	False Negative
FP	False Positive
FROC	Free-response Receiver Operating Characteristic
GT	Ground Truth
IoU	Intersection over Union
mAP	mean Average Precision
PCB	Printed Circuit Board
PI	Power Integrity
PR	Precision–Recall
QC	Quality Control
RAM	Random-Access Memory
ROI	Region of Interest
SI	Signal Integrity
SSIM	Structural Similarity Index
TN	True Negative
TP	True Positive
VOC	Visual Object Classes
XML	Extensible Markup Language

## Appendix A. Implementation Details and Parameter Scaling

This appendix describes implementation-faithful computations that affect reproducibility of the proposed method, including template preselection, ROI discretization, component-to-box confidence scoring, and processing-scale parameter scaling.

### Appendix A.1. Discrete ROI Construction from the Area-Avoidance Ratio

The manuscript derives a continuous border thickness *m* from the area-avoidance ratio *p*. The reference implementation applies an explicit discretization and safety clamp.

Given image size W×H and p∈[0,0.95], define the discriminant

(A1) $$\Delta ={\left(W+H\right)}^{2}-4pWH,\;\Delta \leftarrow \max\left(0,\Delta \right),$$

and compute the smaller root

(A2) $$m=\frac{\left(W+H\right)-\sqrt{\Delta }}{4}.$$

The integer padding is then

(A3) $$\mathrm{padPx}=\left\lfloor \max(0,m)\right\rfloor ,$$

followed by a clamp to prevent degenerate inner regions,

(A4) $$\mathrm{padPx}\leftarrow \min\left(\mathrm{padPx},\;\left\lfloor \frac{\min(W,H)}{2}\right\rfloor -1\right).$$

The resulting ROI is the axis-aligned inner rectangle

(A5) Ω={(u,v):1+padPx≤u≤W−padPx,1+padPx≤v≤H−padPx},

and pixels outside Ω are excluded from thresholding and post-processing.

### Appendix A.2. Template Preselection Score and Shortlist Logic

To accelerate template selection, the implementation uses a cheap preselection score computed for all templates at a small working resolution and then refines SSIM only on a shortlist.

In the reference implementation, the preselection images are computed at max long-side $D_{\mathrm{pre}}$ and are restricted to the same central region Ω used elsewhere: pixels outside Ω are either set to zero (masking) or excluded by cropping, depending on whether SSIM is evaluated by masking or ROI-cropping.

Let $I_{\mathrm{pre}}$ and $T_{k,\mathrm{pre}}$ denote the preprocessed (grayscale, normalized, and resized) query and template images at the preselection resolution. Two preselection scores are supported:

(A6) $$\begin{array}{cc} \mathrm{mad}(I_{\mathrm{pre}},T_{k,\mathrm{pre}}) & =-\frac{1}{n}\sum_{p=1}^{n}|I_{\mathrm{pre}}\left(p\right)-T_{k,\mathrm{pre}}\left(p\right)|, \end{array}$$

(A7) $$\begin{array}{cc} \mathrm{corr}(I_{\mathrm{pre}},T_{k,\mathrm{pre}}) & =\frac{\langle I_{\mathrm{pre}}-\mu_{I},\;T_{k,\mathrm{pre}}-\mu_{T}\rangle }{{∥I_{\mathrm{pre}}-\mu_{I}∥}_{2}\;{∥T_{k,\mathrm{pre}}-\mu_{T}∥}_{2}+\epsilon }, \end{array}$$

where *p* indexes vectorized pixels, *n* is the number of pixels, and $\mu_{I},\mu_{T}$ are the mean pixel values of the respective preselection images.

Both scores are maximized: corr is a normalized (mean-subtracted) correlation, while mad is defined as the negative mean absolute difference so that smaller pixel-wise deviations yield larger scores. The stabilizer ϵ prevents numerical issues when the variance is near zero.

The shortlist is defined as the index set of the top-$K_{s}$ templates under the chosen score,

(A8) $$K_{s}=\mathrm{TopK}\left({\left\{\mathrm{score}\left(I_{\mathrm{pre}},T_{k,\mathrm{pre}}\right)\right\}}_{k=1}^{K},\;K_{s}\right),$$

and SSIM refinement is then computed only for $k\in K_{s}$ on the selection resolution and inner region Ω.

The computational complexity of template preselection can be summarized as follows. Let *K* be the number of templates, and let $P_{\mathrm{pre}}$ denote the number of evaluated pixels in Ω at the preselection resolution; using the common square-grid proxy, $P_{\mathrm{pre}}=O\left(D_{\mathrm{pre}}^{2}\right)$. Computing either mad or corr for a single template is linear in $P_{\mathrm{pre}}$, so scoring all templates is

(A9) $$O\left(K\;P_{\mathrm{pre}}\right)=O\left(K\;D_{\mathrm{pre}}^{2}\right).$$

Constructing the shortlist can be implemented either by (i) sorting all *K* scores, which costs O(KlogK), or (ii) using a top-$K_{s}$ selection routine (e.g., partial sort/heap/selection), which costs $O(K\log K_{s})$ (or O(K) expected time for selection-based methods) when $K_{s}\ll K$. The subsequent SSIM refinement cost is dominated by evaluating SSIM on $K_{s}$ candidates at selection resolution, i.e.,

(A10) $$O(K_{s}\;D_{\mathrm{sel}}^{2}),$$

with smaller constants when SSIM is computed on the cropped ROI Ω rather than on the full image. In practice, grayscale conversion and resizing of templates can be cached to amortize per-query preprocessing and reduce I/O overhead.

### Appendix A.3. Processing Resolution Presets and Parameter Scaling

To reduce runtime on high-resolution images, the implementation optionally runs the core localization stages on a downsampled processing grid and maps outputs back to the original resolution.

Let *L* be the maximum long-side length among the query and selected template, i.e.,

(A11) $$L=\max(H_{I},W_{I},H_{T},W_{T}).$$

Given a preset target long-side length $L_{\mathrm{target}}$ (e.g., 1280 for “720p”, 854 for “480p”), the isotropic processing scale is

(A12) $$s=\min\left(1,\;\frac{L_{\mathrm{target}}}{L}\right).$$

When s<1, both images are resized by *s* prior to registration, score-map computation, and mask extraction.

To maintain approximately consistent behavior across scales, morphology and area parameters are scaled when enabled. Denoting original parameters $\left(A_{\min},r_{c},r_{d}\right)$ and scaled parameters $\left(A_{\min}^{\prime },r_{c}^{\prime },r_{d}^{\prime }\right)$, the implementation uses

(A13) $$A_{\min}^{\prime }=\max\left(1,\mathrm{round}(A_{\min}\;s^{2})\right),\;r_{c}^{\prime }=\max\left(1,\mathrm{round}(r_{c}\;s)\right),\;r_{d}^{\prime }=\max\left(1,\mathrm{round}(r_{d}\;s)\right),$$

where round(·) denotes rounding to the nearest integer (as in the reference implementation). Finally, predicted boxes are mapped back to the original grid by scaling their [xywh] coordinates by 1/s.

### Appendix A.4. SSIM-Map Implementation Details

The SSIM map is computed using MATLAB R2023a’s SSIM to obtain the per-pixel similarity map (second output). For reproducibility, the reference implementation uses grayscale images normalized to [0,1] (dynamic range L=1) and evaluates SSIM with explicit parameters: DynamicRange = 1, $K_{1}=0.01$, $K_{2}=0.03$, GaussianWeights = true, and WindowSize = 11 (square window). Under MATLAB’s SSIM definition, $C_{1}={\left(K_{1}L\right)}^{2}$ and $C_{2}={\left(K_{2}L\right)}^{2}$, so with L=1 the stabilizing constants are $C_{1}=1\times 10^{-4}$ and $C_{2}=9\times 10^{-4}$. For robustness across MATLAB versions, the reference implementation attempts to call SSIM with the above name-value parameters and fall back to the default SSIM(A,B) if a given option (or per-pixel map output) is unsupported; if the per-pixel map output is unavailable, it falls back to using the scalar SSIM value to form a constant map.

### Appendix A.5. Detection Confidence Score from Connected Components

The implementation forms detections by connected components of the binary mask and assigns each component a confidence score computed from the defect-likelihood map values within that component.

Let *Y* be the final binary mask. Let ${\left\{C_{i}\right\}}_{i=1}^{N_{C}}$ denote the connected components of *Y*. Let *M* denote the defect-likelihood (score) map used for thresholding. Each component yields an axis-aligned bounding box $b_{i}$ and a scalar confidence score $s_{i}$ computed by a configurable statistic over $\left\{M\left(u,v\right):\left(u,v\right)\in C_{i}\right\}$. The default implementation uses a robust quantile score:

(A14) $$s_{i}=Q_{q_{s}}\left(\{M\left(u,v\right):\left(u,v\right)\in C_{i}\}\right),\;q_{s}=0.95.$$

For completeness, the implementation also supports $s_{i}=\max_{\left(u,v\right)\in C_{i}}M\left(u,v\right)$ or $s_{i}=\frac{1}{|C_{i}|}\sum_{\left(u,v\right)\in C_{i}}M\left(u,v\right)$.

### Appendix A.6. Data Availability

The dataset and code availability are summarized in the Data Availability statement in the main text. If access issues arise during peer review, the code and evaluation scripts can also be provided by the corresponding author upon reasonable request.

For reproducibility, the experiments in this paper use the PKU-Market-PCB dataset [3] with the following dataset organization under dev/PCB_DATASET/:

- Defect-free template images: PCB_USED(Defect-free)/;
- Defective query images: images(Defective)/ (organized by defect type, e.g., Missing_hole, Mouse_bite, Open_circuit, Short, Spur, Spurious_copper);
- Bounding-box annotations: Pascal VOC XML files under Annotations/ with the same defect-type subfolder structure as images(Defective)/.

### Appendix A.7. Implementation Configuration Used in Experiments

For reproducibility, Table A1 summarizes the key configuration values used to generate the reported results and figures in this manuscript using the mathematical notation introduced in the main text and Appendix B.2. The rightmost column provides the corresponding configuration field in defaultConfig() (dev/v0_14_eval2_release1.m).

**Table A1:** Experiment configuration in manuscript notation (mapped to implementation cfg).

Symbol	Value Used in Experiments	Implementation Mapping
*p*	p=0.07 (area-avoidance ratio for ROI border exclusion)	cfg.roi.avoidAreaRatio (with cfg.roi.enable = true)
*q*	q=0.995 (threshold quantile on Ω for mask extraction)	cfg.mask.quantile
$A_{\min}$	$A_{\min}=150$ px (minimum connected-component area)	cfg.mask.minAreaPx (scaled when resizing is enabled)
$r_{c}$	$r_{c}=6$ px (morphological closing radius)	cfg.mask.closeRadiusPx (scaled when resizing is enabled)
$r_{d}$	$r_{d}=2$ px (morphological dilation radius)	cfg.mask.dilateRadiusPx (scaled when resizing is enabled)
$q_{s}$	$q_{s}=0.95$ (component confidence quantile)	cfg.bbox.scoreMethod = ‘quantile’, cfg.bbox.scoreQuantile = 0.95
*w*	w=0.35 (absolute-difference weight in $M=\left(1-\mathrm{SSIMmap}\right)+w\;D_{\mathrm{abs}}$)	cfg.diff.absDiffWeight (with cfg.diff.useAbsDiff = true)
$\Delta_{\min}$	$\Delta_{\min}=0.0$ (accept alignment iff SSIM gain $\Delta \geq \Delta_{\min}$ on Ω)	cfg.registration.validateTransform = true, cfg.registration.minSSIMGain = 0.0
$\tau_{\mathrm{IoU}}$	$\tau_{\mathrm{IoU}}=0.10$ (IoU threshold for TP/FP/FN matching)	cfg.eval.iouThreshold = 0.1
$D_{\mathrm{pre}}$	$D_{\mathrm{pre}}=128$ px (max long-side for preselect score)	cfg.templateSelect.preselect.maxDim
$K_{s}$	$K_{s}=3$ (shortlist size after preselect)	cfg.templateSelect.preselect.topK
$D_{\mathrm{sel}}$	$D_{\mathrm{sel}}=426$ px (max long-side for SSIM refinement; preset “240p”)	cfg.templateSelect.downscalePreset = ‘240p’
$D_{\mathrm{proc}}$	$D_{\mathrm{proc}}=1280$ px (max long-side for processing; preset “720p”)	cfg.resize.enable = true, cfg.resize.preset = ‘720p’
$\tau_{\theta }$	translation-only transform model for alignment	cfg.registration.type = ‘translation’
ROI eval	SSIM/refinement computed on cropped ROI Ω (faster than masking)	cfg.templateSelect.useROICropForSSIM = true

## Appendix B. Evaluation and Metric Definitions

This appendix collects explicit metric definitions and evaluation-protocol computations used to produce the reported PR/AP/FROC results under the adopted IoU matching protocol.

### Appendix B.1. Operating-Point Threshold Selection (Option A: Best F1)

This paper reports a single reference operating point using Option A (best F1). This operating point is selected from the confidence-ranked detections produced by the pipeline.

Let ${\left\{\left(s_{i},y_{i}\right)\right\}}_{i=1}^{N}$ denote all detections across the evaluated dataset, where $s_{i}\in R$ is the confidence score, and $y_{i}\in \left\{0,1\right\}$ indicates whether the detection is a TP under the adopted per-image greedy one-to-one IoU matching (with $y_{i}=1$ for TP, and $y_{i}=0$ for FP). Let $N_{\mathrm{GT}}$ be the total number of ground-truth boxes across the evaluated images.

Sort detections by score in descending order, producing an ordering π such that $s_{\pi (1)}\geq s_{\pi (2)}\geq \cdots \geq s_{\pi (N)}$. Define the cumulative counts

(A15) $$\mathrm{TP}\left(k\right)=\sum_{i=1}^{k}y_{\pi (i)},\;\mathrm{FP}\left(k\right)=\sum_{i=1}^{k}\left(1-y_{\pi (i)}\right),\;k=1,\ldots ,N.$$

The corresponding precision, recall, and F1 sequences are

(A16) $$\begin{array}{cc} P(k) & =\frac{\mathrm{TP}(k)}{\mathrm{TP}(k)+\mathrm{FP}(k)+\epsilon }, \end{array}$$

(A17) $$\begin{array}{cc} R(k) & =\frac{\mathrm{TP}(k)}{N_{\mathrm{GT}}+\epsilon }, \end{array}$$

(A18) $$\begin{array}{cc} F1(k) & =\frac{2\;P(k)\;R(k)}{P(k)+R(k)+\epsilon }. \end{array}$$

The best-F1 index is $k^{*}=\arg\max_{k}F1\left(k\right)$, and the operating-point threshold is chosen as

(A19) $$t^{*}=s_{\pi (k^{*})}.$$

All detections with $s_{i}\geq t^{*}$ are retained to compute the reported operating-point counts and metrics.

For the cross-validated operating-point estimates reported in the main text, we use K=5 folds. Evaluated images (those with available Pascal VOC ground-truth XML) are randomly permuted using a fixed implementation seed and assigned to folds in a round-robin manner; in each fold, $t^{*}$ is selected on the calibration folds and metrics are computed on the held-out fold.

### Appendix B.2. FROC Computation and Image-Level Recall

The evaluation exports FROC curves by sweeping a finite set of score thresholds derived from the score distribution.

Let $S={\left\{s_{i}\right\}}_{i=1}^{N}$ be the set of all detection scores across the evaluated dataset. This work defines a set of candidate thresholds by sampling quantiles of S. Let $Q=\{0,\frac{1}{49},\ldots ,1\}$ (50 evenly spaced quantile levels) and define

(A20) $$T=\mathrm{unique}\left(\{Q_{q}\left(S\right):q\in Q\}\right),$$

then sort T in descending order.

For each threshold t∈T, this work retains detections with $s_{i}\geq t$ and compute dataset-level counts TP(t),FP(t),FN(t) by performing greedy one-to-one matching within each image (each ground-truth box matches at most one detection and vice versa) under the adopted IoU threshold. Let $N_{\mathrm{img}}$ denote the number of evaluated images that have available ground-truth annotations, and let $N_{\mathrm{GT}}$ denote the total number of ground-truth boxes across those images.

The object-level FROC quantities are

(A21) $$\mathrm{FP}/\mathrm{image}\left(t\right)=\frac{\mathrm{FP}(t)}{N_{\mathrm{img}}+\epsilon },\;{\mathrm{Recall}}_{\mathrm{obj}}\left(t\right)=\frac{\mathrm{TP}(t)}{N_{\mathrm{GT}}+\epsilon }.$$

Image-level recall is computed by counting images that contain at least one ground-truth box and checking whether any TP occurs in the image at threshold *t*. Let $N_{\mathrm{img}}^{\mathrm{GT}}$ be the number of images with at least one ground-truth box. Let $N_{\mathrm{img}}^{\mathrm{TP}}\left(t\right)$ be the number of those images for which at least one retained detection is matched as a TP at threshold *t*. Then

(A22) $${\mathrm{Recall}}_{\mathrm{img}}\left(t\right)=\frac{N_{\mathrm{img}}^{\mathrm{TP}}\left(t\right)}{N_{\mathrm{img}}^{\mathrm{GT}}+\epsilon }.$$

In the manuscript, FROC plots use ${\mathrm{Recall}}_{\mathrm{obj}}\left(t\right)$ versus FP/image(t); ${\mathrm{Recall}}_{\mathrm{img}}\left(t\right)$ is reported for completeness.

### Appendix B.3. IoU for [x y w h] Boxes

Let b=[xywh] and $g=[x^{\prime }\;y^{\prime }\;w^{\prime }\;h^{\prime }]$. Define corners $\left(x_{1},y_{1}\right)=\left(x,y\right)$, $\left(x_{2},y_{2}\right)=\left(x+w,y+h\right)$ and similarly for *g*. The intersection width/height are

(A23) $$\begin{array}{cc} w_{I} & =\max\left(0,\min\left(x_{2},x_{2}^{\prime }\right)-\max\left(x_{1},x_{1}^{\prime }\right)\right), \end{array}$$

(A24) $$\begin{array}{cc} h_{I} & =\max\left(0,\min\left(y_{2},y_{2}^{\prime }\right)-\max\left(y_{1},y_{1}^{\prime }\right)\right), \end{array}$$

with intersection area $A_{I}=w_{I}h_{I}$. The IoU is

(A25) $$\mathrm{IoU}\left(b,g\right)=\frac{A_{I}}{A\left(b\right)+A\left(g\right)-A_{I}+\epsilon }.$$

### Appendix B.4. Average Precision from Confidence-Ranked Detections

Let all detections across the dataset be ranked by confidence *s* in descending order. For a fixed IoU threshold, each detection is labeled TP or FP by greedy matching within each image (each ground-truth box may match at most one detection). Let TP(t) and FP(t) be cumulative counts up to rank *t*, and let $N_{\mathrm{GT}}$ be the total number of ground-truth boxes. Precision and recall sequences are

(A26) $$P\left(t\right)=\frac{\mathrm{TP}(t)}{\mathrm{TP}(t)+\mathrm{FP}(t)+\epsilon },\;R\left(t\right)=\frac{\mathrm{TP}(t)}{N_{\mathrm{GT}}+\epsilon }.$$

AP is the area under the precision–recall curve. This work uses a standard VOC-style discrete approximation based on the precision envelope $\tilde{P}\left(r\right)=\max_{\hat{r}\geq r}P\left(\hat{r}\right)$ and piecewise integration over recall. In our experiments, AP is computed using the all-points VOC interpolation (precision envelope with integration over all recall steps), not the VOC 2007 11-point variant.
